# Preparation of *Komagataeibacter xylinus* Inoculum for Bacterial Cellulose Biosynthesis Using Magnetically Assisted External-Loop Airlift Bioreactor

**DOI:** 10.3390/polym13223950

**Published:** 2021-11-15

**Authors:** Anna Żywicka, Daria Ciecholewska-Juśko, Radosław Drozd, Rafał Rakoczy, Maciej Konopacki, Marian Kordas, Adam Junka, Paweł Migdał, Karol Fijałkowski

**Affiliations:** 1 Department of Microbiology and Biotechnology, Faculty of Biotechnology and Animal Husbandry, West Pomeranian University of Technology in Szczecin, Piastów Ave. 45, 70-311 Szczecin, Poland; daria.ciecholewska@zut.edu.pl (D.C.-J.); radoslaw.drozd@zut.edu.pl (R.D.); 2 Department of Chemical and Process Engineering, Faculty of Chemical Technology and Engineering, West Pomeranian University of Technology in Szczecin, Piastów Ave. 42, 71-065 Szczecin, Poland; rafal.rakoczy@zut.edu.pl (R.R.); maciej.konopacki@zut.edu.pl (M.K.); marian.kordas@zut.edu.pl (M.K.); 3 Department of Pharmaceutical Microbiology and Parasitology, Faculty of Pharmacy, Medical University of Wroclaw, Borowska 211a, 50-534 Wrocław, Poland; adam.junka@umed.wroc.pl; 4 Department of Environment, Hygiene and Animal Welfare, Faculty of Biology and Animal Science, Wroclaw University of Environmental and Life Sciences, Chełmońskiego 38C, 51-630 Wrocław, Poland; pawel.migdal@upwr.edu.pl

**Keywords:** rotating magnetic field, airlift bioreactor, inoculum, bacterial cellulose, fermentation

## Abstract

The aim of this study was to demonstrate the applicability of a novel magnetically assisted external-loop airlift bioreactor (EL-ALB), equipped with rotating magnetic field (RMF) generators for the preparation of *Komagataeibacter* *xylinus* inoculum during three-cycle repeated fed-batch cultures, further used for bacterial cellulose (BC) production. The fermentation carried out in the RMF-assisted EL-ALB allowed to obtain an inoculum of more than 200× higher cellular density compared to classical methods of inoculum preparation. The inoculum obtained in the RMF-assisted EL-ALB was characterized by a high and stable metabolic activity during repeated batch fermentation process. The application of the RMF-assisted EL-ALB for *K. xylinus* inoculum production did not induce the formation of cellulose-deficient mutants. It was also confirmed that the ability of *K. xylinus* to produce BC was at the same level (7.26 g/L of dry mass), regardless of inoculum age. Additionally, the BC obtained from the inoculum produced in the RMF-assisted EL-ALB was characterized by reproducible water-related properties, mechanical strength, nano-fibrillar structure and total crystallinity index. The lack of any negative impact of inoculum preparation method using RMF-assisted EL-ALB on BC properties is of paramount value for its future applications, including use as a biomaterial in tissue engineering, wound healing, and drug delivery, where especially BC liquid capacity, nanostructure, crystallinity, and mechanical properties play essential roles.

## 1. Introduction

Bacterial cellulose (BC) is a polysaccharide polymer synthesized by a variety of bacteria, including non-pathogenic *Komagataeibacter* genus (formerly known as *Gluconacetobacter*). BC has attracted significant interest thanks to its unique physical and chemical properties. Such characteristics as high tensile strength, extremely hydrophilic surface, unique nanostructure, excellent biodegradability, and biological affinity make BC a promising material in a broad spectrum of applications, including medicinal dressings and food production, to name just a few [1,2,3,4]. Nevertheless, a wide application of BC depends on such practical considerations as the scaling-up capability and process cost reduction.

The current methods of BC production are based mainly on static and agitated cultures, as well as various types of airlift bioreactors. Regardless of the type of culturing, the biosynthesis process is carried out as semi-continuous, continuous, or fed-batch fermentation and it must always be preceded by inoculum preparation [5,6,7]. Inoculum preparation is a process in which dormant microbial cells are introduced from a stock culture to a favorable environment and grow to form a metabolically active microbial population. Such cells can be used for inoculation in the productive stage in a bioreactor [7]. An appropriate inoculum of microorganisms consists of a sufficient volume of a metabolically active culture retaining its product-forming ability [8,9,10]. Inoculum preparation is an extremely important stage in every biotechnological process using live cells and it directly affects the efficiency of fermentation. According to Webb and Kamat, the variability in the yield and productivity of the fermentation process can be attributed to a poorly controlled process of initial inoculation [11]. In other words, the quantity and quality of the inoculum have a significant impact on the quantity and quality of the final product [12,13]. Although the above statement also concerns the production of BC, with the exception of a single scientific publication by Wang et al., there are presently no reports on the optimization of *K. xylinus* inoculum preparation or comparisons of inoculum preparation methods [14].

An inoculum of *K. xylinus* is most often prepared in a static or shaken culture [14,15,16]. Both methods have significant limitations, especially when the inoculation concerns several thousand liters of the fermentation medium (the volume applied in industrial-scale BC production). The static culture method results in the accumulation of a gelatinous membrane produced at the air-liquid interface. After incubation, in order to remove the cells embedded in the BC, the membrane is vigorously shaken or homogenized, or enzymatically digested [15,16,17]. The obtained cell suspension can then be used as an inoculum in another fermentation process. However, such methods provide insufficient inoculum volume of cells with a relatively low metabolic activity. As reported by Hornung et al., only some of the total number of cells which are immobilized within the aerobic zone (of approximately 1 mm in thickness), are able to produce BC [16]. A previous study performed by our research group confirmed that due to the limited availability of oxygen in the deeper layers of the BC membrane, the number of living cells was relatively low as compared to its top layer [18]. During static culturing, the floating cellulose pellicle becomes a barrier for the glucose and oxygen transfer in the later stages of its synthesis [16]. Therefore, bacteria in deeper zones are inactive and cannot synthetize BC. Equally important is the time needed to prepare a sufficient amount of inoculum, which in the case of static culture is relatively long and lasts at least a few (usually 7–10) days [15,18].

In contrast to static cultures, the main advantage of an agitated culture for inoculum preparation is a high bacterial cell concentration thanks to the increased rate of transfer of substrates and oxygen. Therefore, shaken culture requires less time to obtain a high number of bacterial cells [19]. However, intensive agitation and aeration of a *K. xylinus* culture can significantly affect the induction of a spontaneous transformation of cellulose-producing bacteria into non-cellulose-producing (Cel^−^) mutants, thereby significantly reducing the efficiency of the production process. Despite these disadvantages, the agitated method is the only one that can provide a sufficient volume and density of *K. xylinus* biomass, needed for an industrial-scale BC production [14,19].

The use of various types of force fields (e.g., magnetic, electrical, ultrasound) is a promising alternative to traditional methods of intensification of biomass production. It was already shown that static magnetic fields (SMF) display a high potential for stimulating the growth of certain microbial species and for increasing the level of enzyme production [20,21,22]. In turn, recent reports of our team and other researchers indicate the applicability of the rotating MF (RMF) for various biotechnological processes, including BC biogenesis [23,24,25,26,27].

In our previous studies, we have proved that exposure to the RMF causes a significant increase in growth and metabolic activity of such versatile microorganisms as *Staphylococcus aureus, Streptococcus mutans*, *Staphylococcus xylosus*, *Escherichia coli, Serratia marcescens, Cronobacter sakazakii,* and *Klebsiella oxytoca* [28,29]. Similar results were obtained for different strains of *K. xylinus* capable of producing BC [24,25,26,27]. Additionally, the application of RMF-assisted bioreactors (described in our previous studies) allowed to significantly increase the BC yield in comparison with a conventional static process [24,25,26,27,28,29]. Noteworthy, the application of the RMF did not increase the number of mutants unable to produce cellulose within the exposure time of 72 h [26]. It was also found that the observed effect was correlated with the time of magnetic exposure during *K. xylinus* cultivation [30].

The hypothesis behind the current study was that the application of an RMF-assisted bioreactor enhances the quality and quantity of *K. xylinus* inoculum, which translates into high yield of the obtained BC polymer. The experiments of our and other research teams performed so far concerned the application of RMF-assisted bioreactors only in laboratory scale due to unresolved technological and operational constraints, related directly to the RMF generator [26,28,29,30,31]. Due to these constraints, the RMF-assisted bioreactors presented in previous studies allowed to conduct the process of BC production in tanks of no more than 3 L volume. It should be noted that our previous studies included the production process of the BC and were conducted only in a stationary culture, with a maximum volume of medium 100 mL, and the exposure time to RMF was not longer than 72 h. Additionally, the inoculum that was used for the culture was not exposed to RMF before the experiment. In the current study we present the newly developed magnetically assisted external-loop airlift bioreactor (EL-ALB), equipped with the RMF generators, enabling *K. xylinus* inoculum development in a significantly increased (up to 100 L) volume of the process chamber.

Performed prior to this study, the analyses of fluid hydrodynamics showed the enhancement of such hydrodynamic parameters as downcomer liquid velocity, mean liquid circulation velocity, mean circulation time and mixing time in RMF-assisted EL-ALB in comparison with an EL-ALB without the application of the RMF [32]. These results suggest that the application of the RMF-assisted EL-ALB could improve the mass transfer rate during the fermentation process and thus increase the rate of *K. xylinus* cell multiplication. Therefore, the aim of this study was to demonstrate the applicability of the novel magnetically assisted external-loop airlift bioreactor for the preparation of *K. xylinus* inoculum in repeated batch fermentation process, subsequently used for BC production.

## 2. Materials and Methods

### 2.1. Experimental Apparatus

**Figure 1:**
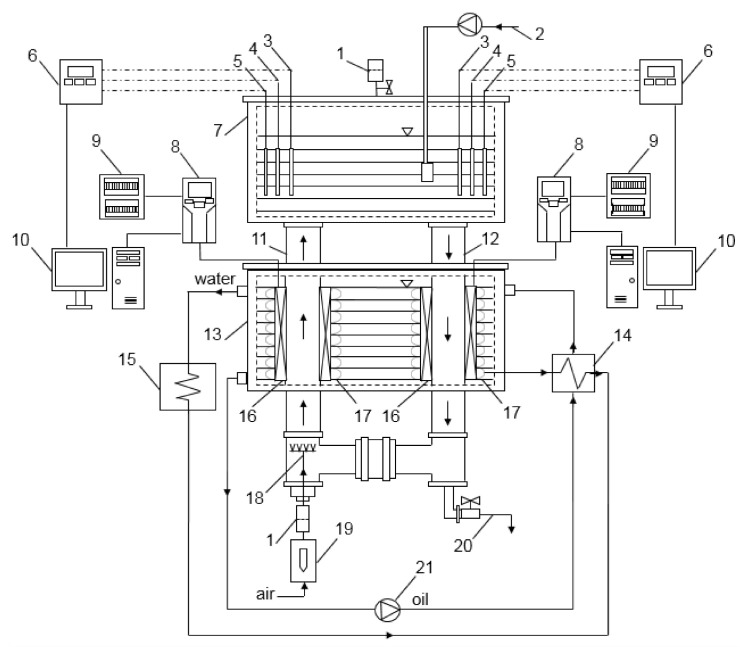
Experimental set-up: 1—filters; 2—fermentation medium dosing system; 3—temperature probes; 4—pH probes; 5—DO% probes; 6—measuring and control equipment; 7—upper chamber (separation zone); 8—inverters; 9—electrical boxes; 10—computers; 11—rising column; 12—falling column; 13—cooling liquid chamber; 14—heat exchanger; 15—thermostat; 16—magnetic field generators; 17—coils; 18—sparger; 19—rotameter; 20—inoculum removal system; 21—circulation pump.

For the production of inoculum, a magnetically assisted external-loop airlift bioreactor (EL-ALB) equipped with RMF generators was used. The experimental apparatus consists of a cooling liquid chamber and upper chamber (separation zone), rising and falling columns, magnetic field generator and the necessary measurement equipment and installations (Figure 1) [32]. The concept and design of RMF-assisted EL-ALB was based on the results of our previous research [24,25,26,27].

### 2.2. Inoculum Preparation in RMF-Assisted EL-ALB

The inoculum preparation was carried out during three-cycle repeated fed-batch cultures using *Komagataeibacter* *xylinus* ATCC 53524 (American Type Culture Collection) strain. A standard culture medium for BC production Hestrin–Schramm (H-S) was used for fermentation (glucose—2 *w*/*v*%, yeast extract—0.5 *w*/*v*%, bacto-peptone—0.5 *w*/*v*%, citric acid—0.115 *w*/*v*%, Na_2_HPO_4_—0.27 *w*/*v*%, MgSO_4_·7H_2_O—0.05 *w*/*v*% and ethanol—1 *v*/*v*).

**Figure 2:**
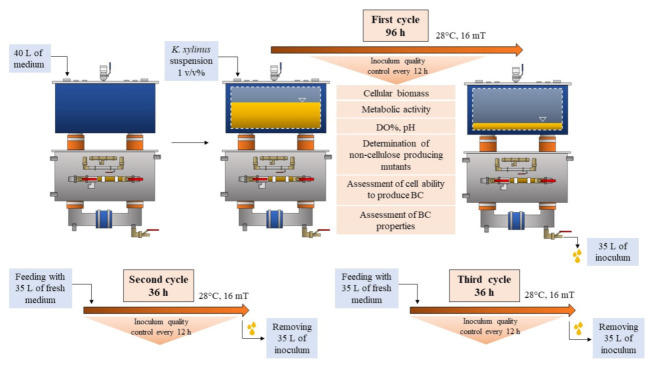
Scheme of the experiment.

The scheme of the experiment is presented in Figure 2. Initially, several colonies of *K. xylinus* were transferred from an agar plate to the H-S medium in order to obtain cell concentration of 2 × 10^5^ colony forming units (CFU mL^−1^). In the first cycle, 1 *v*/*v*% of *K. xylinus* suspension was added to 40 L of a sterile fermentation medium. The inoculum preparation process was carried out in RMF-assisted EL-ALB at 28 °C with constant aeration at the level of 0.05 ννm (the vol. of air/bioreactor working vol. per min). *K. xylinus* cells were exposed to RMF for 12 h in each subsequent 24 h of fermentation. The magnetic induction was equal to 16 mT. The bacterial culture was exposed to the RMF as it passed through the rising and falling columns of the bioreactor system. Exposure parameters were selected based on our previous research, considering the effect of the exposure to the rate of cell proliferation as well as the cost consumption associated with the generation of the magnetic field [28,29,30,31,32].

After the first batch lasting 96 h, 35 L of the medium was removed from the RMF-assisted EL-ALB. Next, the bioreactor was supplemented with a fresh medium to the initial volume (40 L) for the second batch. The second and third batch was carried out for 36 h under the same conditions as described above (Figure 2). The duration of the first fermentation cycle was chosen based on our preliminary study which showed that the number of *K. xylinus* cells reached a maximum value after 96 h of fermentation (after 96 h no further increase was noticed within the next 48 h of incubation) (Supplementary Figure S1). Subsequent fermentation cycles were carried out until the number of *K. xylinus* cells reached the values from the first cycle. The entire process was carried out for 168 h. The first cycle of the fermentation process was also carried out in EL-ALB at the same conditions, but without exposure to the RMF.

### 2.3. Assessment of Fermentation Parameters

During fermentation, the pH of the medium was measured using CX701 Multifunction meter (Elmetron, Zabrze, Poland). Dissolved oxygen (DO%) was also controlled using a ProODO Optical Dissolved Oxygen Instrument (YSI Inc., Beijing, China). Measurements were performed every 12 h.

### 2.4. Evaluation of Inoculum Quality Produced in the RMF-Assisted EL-ALB

#### 2.4.1. Determination of the Number of Living Cells

The number of living cells was determined by quantitative plating. For the test, serial dilutions of the bacterial suspension taken from the bioreactor were spread on Petri dishes containing the H-S agar medium. After 48 h of incubation at 28 °C (Galaxy R PLUS CO2 Incubators, RS Biotech, Irvine, UK), the number of log_10_ CFU mL^−1^ was determined.

#### 2.4.2. Determination of the Cells’ Metabolic Activity

In order to determine the metabolic activity of *K. xylinus* cells, the AlamarBlue (ThermoFisher Scientific, Waltham, MA, USA) assay was performed [33]. For the test, 50 mL of the sample taken from the bioreactor was centrifuged for 20 min at 3300× *g* (Centrifuge 5804 R, Eppendorf, Hamburg, Germany). The resulting pellets were washed in PBS (Phosphate Buffered Saline, MilliporeSigma, Burlington, MA, USA), centrifuged again at 3300× *g* for 20 min and restored to 1 mL of PBS. Next, 100 µL of bacterial suspension was transferred to a 96 well plate (Becton Dickinson and Company, Franklin Lakes, NJ, USA) and 10 µL of AlamarBlue reagent was added to the wells. The plate was incubated for 45 min at 28 °C. The fluorescence signal was measured using microplate fluorescence reader at wavelengths of 530 nm (excitation) and 590 nm (emission).

#### 2.4.3. Determination of Cellulose-Deficient (Cel^−^) Mutants

The number of Cel^−^ mutants was determined in the culture medium taken from the bioreactor during the fermentation process by performing quantitative plating as describe in the section Determination of the number of living cells. Next, the grown colonies were flooded with 20 mL of PBS with 0.01% of Tinopal LPW dye (Calcofluor White M2R, Tinopal UNPA-GX, MilliporeSigma, Burlington, MA, USA) and incubated for 24 h in darkness. The colonies were examined using an UV Cabinet (CAMAG, Muttenz, Switzerland) at a wavelength of 366 nm. BC fibrils stained with a fluorescent dye were observed only around the cellulose-producing colonies (in contrast to the Cel^−^ mutants).

#### 2.4.4. Assessment of the Cells’ Ability to Produce BC

Every 24 h of the fermentation process, 100 mL of the bacterial suspension was taken from the bioreactor and transferred into the Petri dishes (dimensions: 150 × 20 mm) with vents. The BC production was carried out under static conditions for 5 days at 28 °C without any interruptions. After incubation, the BC was harvested from the media and weighed on an analytical balance (WTB 2000, Radwag, Radom, Poland). Next, the BC pellicles were purified by treatment with 0.1 M NaOH (Chempur, Piekary Śląskie, Poland) at 90 °C for 30 min to remove bacterial cells and media components and rinsed with sterile water until the pH become neutral.

### 2.5. Evaluation of the Classical Methods of Inoculum Preparation

In order to compare the effectiveness of inoculum production using the RMF-assisted EL-ALB, the standard stationary and agitated conditions were evaluated. For this purpose, 1 *v*/*v*% of *K. xylinus* suspension at a concentration of 2 × 10^5^ CFU mL^−1^ was added to 100 mL of the H–S medium. The culture was carried out in stationary conditions for 7 days or in agitated conditions for 4 days at 28 °C. Then, the obtained BC was digested with the enzyme cellulase (100 µL/1 mL 0.05 M citrate buffer, pH 4.8, Sigma-Aldrich, Germany). The number of living cells in the bacterial suspension was determined as described in the section Determination of the number of living cells. Additionally, the cells’ ability to produce BC was also analyzed. For this purpose, the bacterial suspension obtained after the digestion of BC with the cellulase enzyme was washed twice in a fresh H–S medium in order to remove the enzyme. Next, the inoculum was restored to 100 mL of the H–S medium and used as described above in the section Assessment of the cells’ ability to produce BC.

### 2.6. Analysis of Physicochemical Properties of BC Depending on the Age of the Inoculum Produced in RMF-Assisted EL-ALB

#### 2.6.1. Evaluation of Mechanical Properties

Purified, wet BC samples were cut into strips of 30 mm length and 10 mm width using a pneumatic press machine (Instron, Norwood, MA, USA). The tensile strength test was performed using the MTS Synergie 100^®^ machine (MTS Systems Corp, Eden Prairie, MN, USA). The measurements were carried out at a speed of 10 mm/min at room temperature. The average values of tensile strength, Young’s modulus and elongation at break were calculated from the stress-strain curves. All measurements were performed in four replicates [18].

#### 2.6.2. Scanning Electron Microscopy Analysis

Purified and wet BC samples were fixed in glutaraldehyde (POCH, Gliwice, Poland) for 7 days and dried in a critical point dryer. Next, the BC samples were sputtered with Au/Pd (60:40) and examined using a scanning electron microscope (SEM, Auriga 60, Zeiss, Oberkochen, Germany). Fibril diameter was analyzed by means of the ImageJ software (NIH, Bethesda, MD, USA).

#### 2.6.3. ATR-FTIR Analysis of BC

The BC sheet samples purified and dried at 60 °C for 12 h were analyzed using Attenuated Total Reflectance-FTIR spectroscopy with ATR-FT-IR spectrometer (Bruker Co., Leipzig, Germany). After normalization of the obtained spectra at 1160 cm^−1^ wavenumber, the total crystallinity index (TCI) of the dry BC samples was determined as the ratio of absorbance values for bands 1370/2900 cm^−1^ [34,35].

#### 2.6.4. Determination of Water Swelling Ratio

Purified and wet BC samples were dried at 60 °C for 6 h to remove water, weighed, immersed in distilled water for 24 h, and weighed again. The swelling ratio as a percent of dry mass (SR%) was then calculated using Equation (1):

SR (%) = (W_w_ − W_d_)/W_d_ × 100 (1)

where: W_w_ is the weight of the swollen BC and W_d_ is the dry weight of the sample.

#### 2.6.5. Determination of Water Holding Capacity

Purified and dried BC samples were weighted, immersed in distilled water for 24 h to obtain maximum absorption level, and weighed again. The ability to hold water was determined using moisture analyzer (Radwag, Radom, Poland) at 60 °C until the weight of BC was equal to the initial value (dry weight before hydration). Weight measurements were made automatically every 2 min. Water holding capacity (WHC) was then calculated using the Equation (2):

WHC = W_rw_/(W_w_ − W_d_) × 100 (2)

where: W_rw_ is the weight of water removed from BC during drying, W_w_ is the initial weight of wet BC, and W_d_ is the dry weight of the sample.

### 2.7. Statistical Analysis

Data was shown as the means ± standard errors of the means (SEM) obtained from three different measurements. Statistical differences between the samples were determined by one-way analysis of variance (ANOVA). All analyses were considered statistically significant when the P value was less than 0.05. The statistical analyses were conducted using Statistica 9.0 (StatSoft, Cracow, Poland).

## 3. Results and Discussion

### 3.1. Measurements of Cellular Parameters

**Figure 3:**
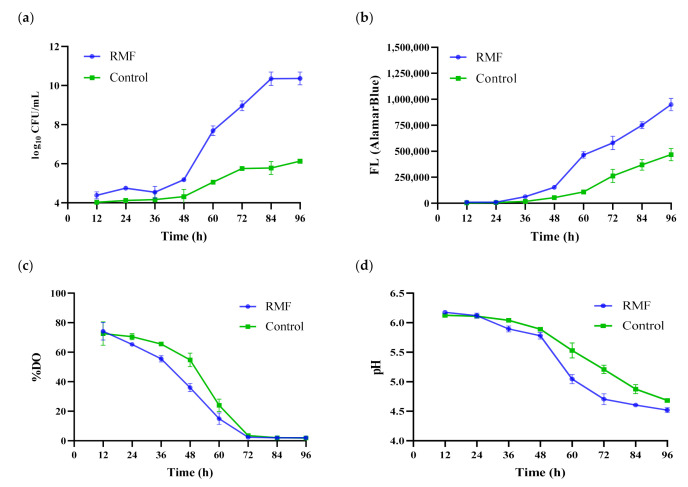
(**a**) The number of living cells; (**b**) metabolic activity of *K. xylinus* based on fluorescence of AlamarBlue reagent; (**c**) pH level; (**d**) %DO during first cycle of fermentation. Data are presented as a mean ± standard error of the mean (SEM).

**Figure 4:**
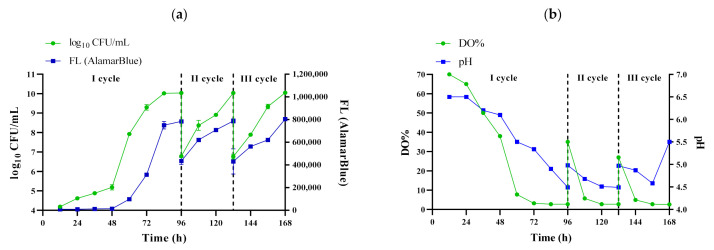
(**a**) The number of living cells and metabolic activity of *K. xylinus* based on fluorescence of AlamarBlue reagent; (**b**) pH and DO% of the culture medium during the process of inoculum preparation. Data are presented as a mean ± standard error of the mean (SEM).

The procedure of inoculum preparation must ensure high density of cells, especially if the process is conducted on an industrial scale [7]. Moreover, the cells should be able to produce a satisfactory and reproducible yield of product [8,10]. The time of inoculum preparation is also an important parameter due to operational costs of the process. In the current study, it was confirmed that exposure to the RMF caused a significant increase in growth of *K. xylinus* cells during first cycle as compared to the fermentation process carried out under the same conditions but without exposure to RMF (Figure 3a). The maximum cell density at the end of the first cycle of the fermentation process (96 h) carried out in EL-ALB with RMF exposure reached 1.14 × 10^10^ CFU mL^−1^ and was approx. 200× higher as compared to the fermentation process (96 h) carried out in EL-ALB without RMF exposure (5.31 × 10^7^ CFU mL^−1^) (Figure 3a). The subsequent fermentation cycles were carried out only in RMF-assisted EL-ALB which aimed to further confirm the stability of the process during repeated fermentation cycles (Figure 4). Second and third fermentation cycle were carried out until the number of *K. xylinus* cells reached the values from the first cycle, which was achieved after 36 h (Figure 4a). There were no statistically significant differences in the cell density obtained in subsequent fermentation cycles.

Additionally, the AlamarBlue assay was performed to investigate the effect of RMF on the *K. xylinus* cell metabolic activity. The AlamarBlue assay incorporates an oxidation-reduction (REDOX) indicator which changes color and fluorinates in response to cell metabolic activity [33]. The results showed that the metabolic activity of *K. xylinus* increased during the first 96 h (Figure 3b). It was revealed that *K. xylinus* cells exposure to RMF showed on average 103% higher metabolic activity as compared to non-exposure cells. Importantly, there were no statistically significant differences in the metabolic activity of the cell suspension in subsequent cycles carried out in RMF-assisted EL-ALB (Figure 4a), which proved the stability of the process during all fermentation cycles.

In our previous reports it was confirmed that RMF has a stimulatory effect on the metabolic activity and growth of *K. xylinus* cells [24,25,26,27]. The stimulatory influences of the exposure to RMF on cellular parameters can relate to the effects exerted by the RMF on bio-liquids. It was proved that RMF causes mixing of the bioliquids at microlevels [30,31,32]. It was demonstrated that the unique feature of RMF is its ability to induce the time-averaged azimuthal force, which drives the flow of the electrically conducting fluid in the azimuthal direction. Thus, the RMF acting on the fluid characterized by electrical conductivity can be considered as an alternative method of mixing [28,29,30,31,32]. In magnetic-susceptible liquid (e.g., H-S medium of the electrical conductivity of approx. 7 mS), an externally applied RMF induces an arbitrary virtual loop which rotates at a frequency equal to the frequency of the supply current. According to the induction law, the electric current density is induced along the loop. The interaction between this vector and the magnetic field generates electromagnetic force that causes movement of the fluid in the direction of the magnetic field spinning. This force is responsible for the mixing effect, which may increase the efficiency of transport processes between the medium and the microorganisms [30,31,32]. It is also thought that the associated currents can be induced in the culture medium in consequence of the magnetic field because the culture medium contains various electrolytes, e.g., Na^+^, K^+^, Mg^2+^, NH^4+^ and their associated anions, e.g., sulphate, phosphate and chlorate, along with microbial cells that contain various components including ionic solutions, proteins and lipids, which are susceptible to the influence of magnetic or induced electric fields.

Additionally, the use of RMF generators improves the downcomer liquid velocity and mean liquid circulation velocity [32]. Moreover, as also proved by Lechowska et al., the mean circulation time and mixing time under the RMF action have significantly lower values in comparison with conventional EL-ALB [32].

It should be mentioned that our previous studies were conducted in static conditions, and with a volume of the culture not exceeding 25 mL. It should be noted that a successful optimization of the process in a laboratory scale does not guarantee an analogical success (with regard to yield, enzymatic activity, etc.) of a pilot-scale or industrial process [36,37].

The optimal process of *K. xylinus* inoculum preparation requires high multiplication of cells and lack or very low production of BC. It is because the freshly formed BC fibers can deposit on the elements of the bioreactor, obstructing air supply, sampling or measurements of temperature, pH and DO% [16,19,38]. The above-mentioned disadvantages can be reduced significantly by the addition to the fermentation medium of enzymes such as various types of microbial cellulases [14]. However, this approach is also limited by the needs for further deactivation of the used enzymes. In the current study, a small amount of BC was produced during the fermentation process, despite the fact that the cell concentration obtained in the RMF-assisted EL-ALB was high. Taking into account the results of our previous study, it may suggest that the application of rising and falling columns in the RMF-assisted EL-ALB improved the hydrodynamic parameters and prevented the formation and deposition of BC [32]. It bestowed the cells with optimal conditions for multiplication without the addition of cellulose-digesting enzymes or inhibitors to the bioreactor.

The results presented in Table 1 confirmed that fermentation carried out in the RMF-assisted EL-ALB allowed to obtain an inoculum with a significantly higher density of *K. xylinus* cells compare to fermentation carried out in EL-ALB without RMF after 96 h. The number of *K. xylinus* cells was more than 200× higher as compared to the bacterial suspension obtained in EL-ALB without RMF. The result also showed that fermentation carried out in the RMF-assisted EL-ALB allowed to obtain an inoculum with a significantly higher density of *K. xylinus* cells with the bacterial suspension obtained after enzymatic digestion. The application of the RMF-assisted EL-ALB allowed to obtain more than 300× and 8000× higher cell density in the same volume of the H-S medium (100 mL) as compared to the bacterial suspension obtained after enzymatic digestion of BC biosynthesized in an agitated and static culture, respectively. It should be also noted that the inoculum preparation time in the RMF-assisted EL-ALB was shorter as compared to the bacterial suspension obtained after enzymatic digestion. The process run time directly affects the operating costs of the equipment and energy costs.

The number of cells obtained from BC biosynthesized under agitated conditions was higher as compared to static conditions. The increased number of *K. xylinus* cells obtained in agitation or airlift bioreactors is associated with more oxygen in the medium, as previously confirmed by other authors [16,38,39]. However, the application of airlift bioreactors in a fermentation process provides lower shear stress and shutdowns of the medium, compared to agitated cultures. It should be also noted that the inoculum preparation time in the RMF-assisted EL-ALB was shorter as compared to the bacterial suspension obtained after enzymatic digestion.

### 3.2. Fermentation Parameters-pH and DO%

*K. xylinus* is a strictly aerobic bacterium; thus, an appropriate oxygen supply is crucial for its cultivation [16]. An inadequate mass transfer rate of oxygen limits cell growth and product formation [5,40]. Figure 4b presents changes in the DO% in the medium during fermentation carried out in RMF-assisted EL-ALB in subsequent cycles. The results showed a substantial decrease in the DO% concentration during the first 48 h of fermentation. After 48 h of incubation, DO% remained at relatively similar level (on average 3%) until the end of the first cycle. Similar results were obtained for the fermentation process carried out in EL-ALB without RMF exposure (Figure 3c).

The results obtained in subsequent cycles showed that DO% increased to 35% and 30%, respectively, in the second and third cycle of fermentation carried out in RMF-assisted EL-ALB, after the addition of 35 L of fresh medium. However, the level of DO% decreased rapidly to 3% during the next 24 h in the second and third cycle (Figure 4b).

Despite the decrease in DO%, the growth rate of *K. xylinus* cells was not affected (Figure 4a vs. Figure 4b). Similar observations were made by Cheng et al. who revealed that despite DO% drop to 2%, no significant decrease in biomass production was observed [41]. Moreover, the above authors also showed that the increased value of oxygen-transfer coefficient (k_L_a) during cultivation in an airlift bioreactor positively influenced the growth rate of *K. xylinus*. Lechowska et al. showed that the k_L_a was significantly increased in an RMF-assisted EL-ALB in comparison with a conventional EL-ALB [32]. It should also be mentioned that increasing the k_L_a is a more economical approach with regard to improving the performance of oxygen transfer than supplying a higher concentration of oxygen in the bioreactor [41].

The pH of the culture medium is one the most limiting factors that can significantly influence *K. xylinus* cell growth and productivity of BC [41,42]. Changes in the pH of the medium are associated mainly with the production by *K. xylinus* cells of gluconic and acetic acid during BC biosynthesis, when glucose and ethanol are used as a sole carbon source [15,16,38]. Optimal pH values range from 4.5 to 7.5, with the greatest efficiency regarding BC production being around 6.5. If the pH drops below 3.5, cellulose synthesis is inhibited. It was demonstrated that the pH value of the fermentation medium decreased steadily, reaching approximately 4.5 after first cycle. There were no statistically significant differences between the pH level during first cycle of fermentation (96 h) carried out in EL-ALB with and without RMF exposure (Figure 3d). It should be noted that during subsequent fermentation cycles carried out in RMF-assisted EL-ALB the pH did not drop below the level which could affect the ability to biosynthesize BC by *K. xylinus* cells; therefore, there was no need to further stabilize the pH (Figure 4b).

### 3.3. Determination of Cellulose-Deficient (Cel^−^) Mutants

As stated above, the microbial inoculum should be able to retain its ability to form a high yield of product. Several authors confirmed that the ability of eukaryotic and prokaryotic cells used in the fermentation processes to produce useful/valuable metabolites may be lost, especially in the case of repeated culture transfers. Therefore, strain stability is one of the major concerns [7,9].

It should also be mentioned that BC is a natural type of biofilm that protects the cells from adverse environmental conditions [43]. Under optimal conditions provided in a bioreactor, a subpopulation of cells may lose its ability to produce BC, with such subpopulations being referred to as Cel^−^ mutants. Several authors reported more frequent appearance of Cel^−^ mutants in agitated cultures with a high oxygen supply and high volumetric agitation power compared to static conditions [16,38]. The frequency of mutant occurrence is one of the most important factors lowering the quality of inoculum produced in shaken cultures.

**Figure 5:**
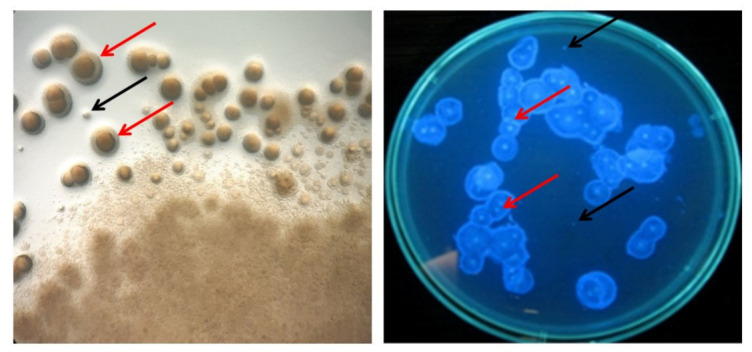
Visualization of cellulose producing bacteria and non-producing mutants. A red arrow marks the cellulose around the *K. xylinus* colony. A black arrow marks the colony of non-producing mutants.

During three cycles of fermentation, the number of *K. xylinus* colonies which were unable to synthesize cellulose was on average 1.43 × 10^4^ CFU mL^−1^, which accounted for approximately 0.00012% of all *K. xylinus* cells present in the culture (Table 2). As can be seen in Figure 5, luminescent zones were observed around the cellulose-producing colonies. There were no statistically significant differences between the number of Cel^−^ mutants after first cycle of fermentation (96 h) carried out in EL-ALB with and without EMF exposure (Table 2). These findings stay in line with our previous result which reviled that the application of the RMF did not increase the number of mutants unable to produce cellulose within the exposure time (72 h) when the static culture was performed [26].

The number of Cel^−^ mutants which appeared during fermentation in RMF-assisted EL-ALB was lower compared to the results presented by other authors. In the research presented by Park et al., the number of Cel^−^ mutants after 96 h of incubation in agitated conditions (200 rpm) was on average 2 × 10^5^ CFU mL^−1^ [44]. Wang et al. showed that after 96 h of incubation in a shaking culture (120 rpm) the number of Cel^−^ mutants was 4 × 10^6^ CFU mL^−1^ (0.2%) and Aydin and Aksoy found that mutant ratio (number of Cel^−^/number of total cells) increased along with the shaking rate and the number of batches, obtaining a maximum of 2% in the fifth batch at the speed of 200 rpm [14,45].

The results obtained in the current study can be explained by the relatively low DO% level during the process (35–3% of DO) (Figure 4b) being a result of the low air-flow applied during fermentation. The fermentation process in RMF-assisted EL-ALB was carried out with constant aeration at an air-flow rate of 0.05 ννm, which is much lower than the one commonly used for *K. xylinus* cultivation (0.5–2 ννm) [4,46,47].

### 3.4. Assessment of Cell Ability to Produce BC

**Figure 6:**
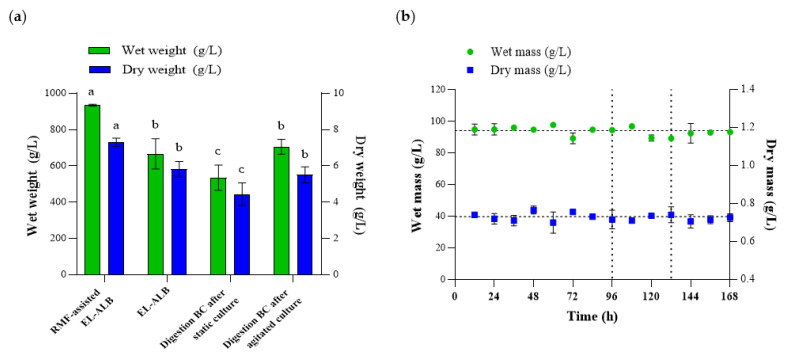
(**a**) The weight of BC depends for the method of inoculum preparation; (**b**) The weight of BC obtained using inoculum produced in RMF-assisted EL-ALB during three-cycle repeated fed-batch cultures. Data are presented as a mean ± standard error of the mean (SEM). a, b, c—statistically significant difference between method of inoculum preparation. The differences were considered statistically significant when the *p*-value was less than 0.05.

The ability to produce BC was determined for the inoculum obtained in the RMF-assisted EL-ALB and after enzymatic digestion of the cellulose membrane obtained in a static or agitated culture. For BC biosynthesis, the same volume of the H–S medium (100 mL) was used. The results showed statistically significant differences in the amount of the obtained BC depending on the method of inoculum production (Figure 6a). The average weight of the BC obtained from an inoculum produced in the RMF-assisted EL-ALB was 940 g/L and 7.26 g/L of wet and dry BC, respectively, and was on average 60% higher as compared to the BC obtained after enzymatic digestion of the cellulose membrane. The results of the current study also confirmed that *K. xylinus* ability to produce BC was at the same level regardless of the age of inoculum produced in RMF-assisted EL-ALB during three-cycle repeated fed-batch cultures (Figure 6b). There were no statistically significant differences in BC weight obtained in subsequent cycles of the fermentation process.

It should be mentioned that there are no statistically significant differences in the amount of biosynthesized BC, regardless of the number of cells in the inoculum obtained in the RMF-assisted EL-ALB (Figure 4a vs. Figure 6b). This can be explained by the mechanism of cellulose membrane formation in a stationary culture. BC formation within a static surface culture starts with the formation of island-like cellulose fragments on the broth surface [16]. In the next step, the fragments stick together to form a thin cellulose film. Further incubation results in the accumulation of a gelatinous membrane produced at the air-liquid interface, which becomes a barrier for the glucose and oxygen transfer in subsequent stages of its synthesis. At this point of the process, the initial cell concentration in the medium is no longer relevant. Therefore, in line with the available literature data, it can be stated that a higher density of *K. xylinus* cells in the inoculum determines BC formation rate, however only at the beginning of the process [16,38].

It should be also mentioned that the quantity and quality of the inoculum have a significant impact on the quantity and quality of the final product [11,12,13]. The optimal inoculum size for BC production varies (1–30%) depending on the strain, medium composition, fermentation time and type of culture [4,16,48]. The larger inoculum size with active seed culture minimizes the length of adaptation (lag period) phase and facilitates the biomass concentration with a short fermentation time leading to higher production of exopolysaccharides such as BC [48]. However, if the inoculum concentration is in excess, there would be competition between the cells in utilizing nutrients, which disrupts the bacterial growth and thereby reduces the production of BC [4,49,50]. The rapid consumption of nutrients contained in the medium at larger inoculum size reduces the efficiency of fermentation [48]. On the other hand, if the inoculum size goes below a certain level, the number of bacterial cells in fermentation medium responsible for BC elaboration is minimum, and hence the efficiency of fermentation will be low [49]. Additionally, when the density of the inoculum cells is too low, the time of the adaptation and formation phase of BC is longer.

### 3.5. Analysis of Physicochemical Properties of BC

The parameters of the inoculum used in the biotechnological process determine the quantity and quality of the resulting product, as well as the duration of the process and the operating costs [7]. It has been reported that inoculum age may affect the formation of BC and its properties [49,50]. Therefore, in the current study, the physicochemical properties of BC obtained using the inoculum prepared in the RMF-assisted EL-ALB were analyzed.

**Figure 7:**
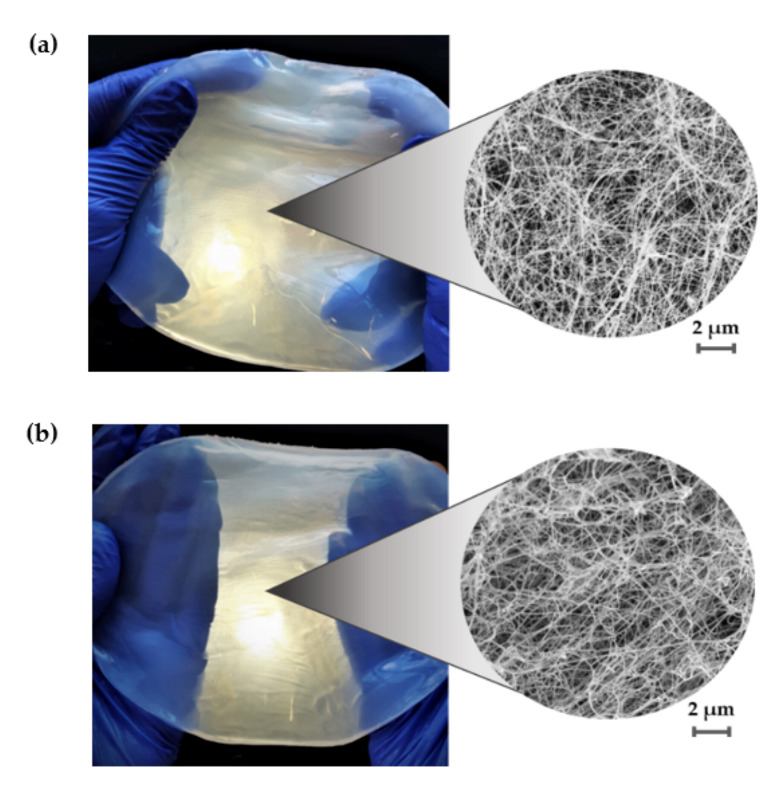
Macromorphology and micromorphology of purified BC obtained from *K. xylinus* ATCC 53524 culture using inoculum produced in RMF-assisted EL-ALB after (**a**) 96 h and (**b**) 168 h; magnification 10,000× (SEM, Auriga 60, Zeiss, Oberkochen, Germany).

For many applications, the water-related properties can be considered to be the most important features of BC, because they determine its absorption capacity and ability to retain and release liquid. Overall, our results indicated that water-related parameters were comparable for BC membranes regardless of the age of the inoculum (Table 3). There were no statistically significant differences between results of the %SR and WHC in subsequent fermentation cycles. The %SR was on average 311%; such a value stays in the range reported by other research teams [51,52,53]. In addition, the value of WHC (4.38%), measured in the present study did not differ significantly from the results presented by the other authors [52,53,54] and in our previous analyses [27,55]. Likewise, regardless of the fermentation cycles, we observed no significant differences in tensile mechanical properties, nano-fibrillar structure and TCI (Table 3). SEM analysis confirmed a presence of coherent 3-D network formed by cellulose fibers (Figure 7) of 53.84 nm average diameter (Table 3, Supplementary Figure S2), whereas in previous report, the range of fibrils’ diameter was 35–70 nm [56]. The average tensile strength, Young’s modulus and elongation at break of BC was 2.41 MPa, 11.75 MPa and 21.04%, respectively. In turn, the FTIR analyses showed that BC displayed a TCI of 1.61 ± 0.26 (Table 3). Likewise, the tensile mechanical properties and TCI values were also comparable with the findings of other authors reported for unmodified BC produced under stationary culture conditions [18,57,58,59,60]. Except for the TCI values, also the ATR-FTIR spectra (Supplementary Figure S3) showed absorption bands characteristic for BC functional groups indicating that the typical cellulose was produced [34,35].

In summary, all of the obtained results were in good agreement with values reported in the literature for typical BC [61,62,63,64,65,66,67,68]. In this context, it should be noted that the physicochemical properties of BC are influenced by many factors, such as the cultivation time and conditions as well as the composition of the culture medium, in addition to the quantity and quality of the inoculum [12,13,14]. Therefore, the lack of any negative impact of inoculum preparation method on BC properties is of paramount value for its future applications, including use as a biomaterial in tissue engineering, wound healing, and drug delivery, where especially BC liquid capacity, nanostructure, crystallinity, and mechanical properties play essential roles.

## 4. Conclusions

In the current study, a novel magnetically assisted external-loop airlift bioreactor (EL-ALB), equipped with RMF generators was used for *K. xylinus* inoculum production during three-cycle repeated fed-batch cultures in the working volume of 40 L. The proposed approach allows to significantly increase cell density as compared to the conventional method. Additionally, *K. xylinus* inoculum was characterized by a high and stable metabolic activity. The application of RMF-assisted EL-ALB did not induce the formation of cellulose-deficient mutants during three-cycle repeated fed-batch cultures. The ability to produce BC was at the same level regardless of inoculum age. Furthermore, the BC obtained using the inoculum produced in the RMF-assisted EL-ALB was characterized by repeatable mechanical strength, nanostructure, and total crystallinity index. The results obtained in this study may find multiple applications in all biotechnological processes requiring a high quality of inoculum.

## Figures and Tables

**Table 1:** Comparison of inoculum preparation methods.

Inoculum Parameters	Enzymatic Digestion BC from Stationary Culture	Enzymatic Digestion BC from Agitated Culture	RMF-Assisted EL-ALB Culture	EL-ALB Culture
**Log_10_ CFU mL^−1^**	5.18 ± 0.76 ^a^	6.76 ± 1.14 ^b^	10.41 ± 1.56 ^c^	7.72 ± 1.25 ^b^
**Time (d)**	7	4	2	2

The results are presented as a mean ± standard error of the mean (SEM). a, b, c—statistically significant difference between methods of inoculum preparation. The differences were considered statistically significant when the *p*-value was less than 0.05.

**Table 2:** Total number of *K. xylinus* cells and cellulose-deficient mutants of *K. xylinus* in H–S medium obtained in RMF-assisted EL-ALB and EL-ALB without RMF exposure (control).

		Cycle I	Cycle II	Cycle III
Total number of cells (log_10_ CFU mL^−1^)	RMF-assisted EL-ALB	10.41 ± 1.56 ^a^	10.1 ± 1.88 ^a^	10.07 ± 2.13 ^a^
	EL-ALB	7.72 ± 1.25 ^b^	-	-
Mutants (log_10_ CFU mL^−1^)	RMF-assisted EL-ALB	4.12 ± 0.57 ^a^	4.20 ± 0.42 ^a^	4.15 ± 0.53 ^a^
	EL-ALB	2.24 ± 0.55 ^b^	-	-
Mutants (% of control)	RMF-assisted EL-ALB	0.00012 ^a^	0.00013 ^a^	0.00011 ^a^

The results are presented as a mean ± standard error of the mean (SEM). a, b—statistically significant difference between methods of inoculum preparation. The differences were considered statistically significant when the *p*-value was less than 0.05.

**Table 3:** Selected properties of BC pellicles synthesized by *K. xylinus*.

Properties	Cycle I	Cycle II	Cycle III
SR	311 ± 27	308 ± 31	314 ± 42
WHC	4.41 ± 1.13	4.33 ± 0.98	4.39 ± 0.59
TS	2.68 ± 0.10	2.35 ± 0.16	2.19 ± 0.21
YM	11.32 ± 1.33	12.09 ± 1.98	11.85 ± 2.13
EB	20.54 ± 2.67	21.55 ± 3.11	21.03 ± 2.87
FD	55.75 ± 5.56	51.83 ± 4.89	53.94 ± 4.11
TCI	1.62 ± 0.13	1.57 ± 0.33	1.64 ± 0.21

Data are presented as a mean ± standard error of the mean (SEM). SR—swelling ratio (%); WHC—water holding capacity after 10 min at 60 °C (%); TS—tensile strength (MPa); YM—Young modulus (MPa); EB—elongation at break (%); FD—Fibril diameter (nm); TCI—Total crystallinity index. There were no statistically significant differences between the results obtained in subsequent cycles (*p* < 0.05).

## Data Availability

The data that support the findings of this study are available from the corresponding author upon reasonable request.

## Supplementary Materials

The following are available online at www.mdpi.com/2073-4360/13/22/3950/s1, Supplementary Figure S1: The number of living cells and metabolic activity of K. *xylinus* based on fluorescence of AlamarBlue reagent; Supplementary Figure S2: Nanostructure of BC obtained using inoculum produced in RMF-assisted EL-ALB after (a) I, (b) II, and (c) III cycle of fermentation; Supplementary Figure S3: ATR-FTIR spectra of BC obtained using inoculum produced in RMF-assisted EL-ALB after I, II, and III cycle of fermentation.

## References

[1] Azeredo H., Barud H., Farinas C.S., Vasconcellos V.M., Claro A. (2019). Bacterial cellulose as a raw material for food and food packaging applications. Front. Sustain. Food Syst..

[2] Gorgieva S., Trcek J. (2019). Bacterial Cellulose: Production, modification and perspectives in biomedical applications. Nanomaterials.

[3] Ullah H., Santos H.A., Khan T. (2016). Applications of bacterial cellulose in food, cosmetics and drug delivery. Cellulose.

[4] Wang J., Tavakoli J., Tang T. (2019). Bacterial cellulose production, properties and applications with different culture methods—A review. Carbohydr. Polym..

[5] Chao Y., Sugano Y., Shoda M. (2001). Bacterial cellulose production under oxygen-enriched air at different fructose concentrations in a 50-L, internal-loop airlift reactor. Appl. Microbiol. Biotechnol..

[6] Lin S.P., Hsieh S.C., Chen K.I., Demirci A., Cheng K.C. (2014). Semi-continuous bacterial cellulose production in a rotating disk bioreactor and its materials properties analysis. Cellulose.

[7] Sood S., Singhal R., Bhat S., Kumar A., Murray M.-Y. (2011). Comprehensive Biotechnology.

[8] Nguyen D.N., Ton N.M.N., Le V.V.M. (2009). Optimization of *Saccharomyces cerevisiae* immobilization in bacterial cellulose by ‘adsorption-incubation’ method. Int. Food Res. J..

[9] Stanbury P.F., Whitaker A., Hall S.J. (2017). Principles of Fermentation Technology.

[10] Żywicka A., Junka A., Ciecholewska-Juśko D., Migdał P., Czajkowska J., Fijałkowski K. (2020). Significant enhancement of citric acid production by *Yarrowia lipolytica* immobilized in bacterial cellulose-based carrier. J. Biotechnol..

[11] Webb C., Kamat S.P. (1993). Improving fermentation consistency through better inoculum preparation. World J. Microbiol. Biotechnol..

[12] Blasco L., Kahala M., Tampio E., Vainio M., Ervasti S., Rasi S. (2020). Effect of inoculum pretreatment on the composition of microbial communities in anaerobic digesters producing volatile fatty acids. Microorganisms.

[13] Rajput A.A., Sheikh Z. (2019). Effect of inoculum type and organic loading on biogas production of sunflower meal and wheat straw. Sustain. Environ. Res..

[14] Wang Z.G., Xiang D., Wang X.B., Li C.F. (2016). Preparation of an inoculum of *Gluconacetobacter xylinus* without mutants in shaken culture. J. Appl. Microbiol..

[15] Atwa N.A., El-Diwany A.I., El-Saied H., Basta A.H. (2015). Improvement in bacterial cellulose production using *Gluconacetobacter xylinus* ATCC 10245 and characterization of the cellulose pellicles produced. Egypt. Pharm. J..

[16] Hornung M., Ludwig M., Gerrard A.M., Schmauder H.P. (2006). Optimizing the production of bacterial cellulose in surface culture: Evaluation of substrate mass transfer influences on the bioreaction. Eng. Life Sci..

[17] Lin S.P., Huang Y.H., Hsu K.D., Lai Y.J., Chen Y.K., Cheng K.C. (2016). Isolation and identification of cellulose-producing strain *Komagataeibacter intermedius* from fermented fruit juice. Carbohydr. Polym..

[18] Żywicka A., Junka A., Szymczyk P., Chodaczek G., Grzesiak J., Sedghizadeh P., Fijałkowski K. (2018). Bacterial cellulose yield increased over 500% by supplementation of medium with vegetable oil. Carbohydr. Polym..

[19] Ha J.H., Shah N., Ul-Islam M., Khan T., Park J.K. (2011). Bacterial cellulose production from a single sugar alpha-linked glucuronic acid-based oligosaccharide. Process Biochem..

[20] Kthiri A., Hidouri S., Wiem T., Jeridi R., Sheehan D., Landouls A. (2019). Biochemical and biomolecular effects induced by a static magnetic field in *Saccharomyces cerevisiae*: Evidence for oxidative stress. PLoS ONE.

[21] Muniz J.B., Marcelino M., da Motta M., Schuler A., da Motta M.A. (2007). Influence of static magnetic fields on *S. cerevisae* biomass growth. Braz. Arch. Biol. Technol..

[22] Tomska A., Wolny L. (2010). Enhancement of biological wastewater treatment by magnetic field exposure. Bioresour. Technol..

[23] Drozd R., Rakoczy R., Wasak A., Junka A., Fijałkowski K. (2018). The application of magnetically modified bacterial cellulose for immobilization of laccase. Int. J. Biol. Macromol..

[24] Drozd R., Szymańska M., Żywicka A., Kowalska U., Rakoczy R., Kordas M., Konopacki M., Junka A.F., Fijałkowski K. (2021). Exposure to non-continuous rotating magnetic field induces metabolic strain-specific response of *Komagataeibacter xylinus*. Biochem. Eng. J..

[25] Fijałkowski K., Żywicka A., Drozd R., Niemczyk A., Junka A.F., Peitler D., Kordas M., Konopacki M., Szymczyk P., El Fray M. (2015). Modification of bacterial cellulose through exposure to the rotating magnetic field. Carbohydr. Polym..

[26] Fijałkowski K., Rakoczy R., Żywicka A., Drozd R., Zielińska B., Wenelska K., Cendrowski K., Peitler D., Kordas M., Konopacki M. (2016). Time dependent influence of rotating magnetic field on bacterial cellulose. Int. J. Polym. Sci..

[27] Fijałkowski K., Żywicka A., Drozd R., Junka A.F., Peitler D., Kordas M., Konopacki M., Szymczyk P., Rakoczy R. (2017). Increased water content in bacterial cellulose synthesized under rotating magnetic fields. Electromagn. Biol. Med..

[28] Fijałkowski K., Nawrotek P., Struk M., Kordas M., Rakoczy R. (2013). The effects of rotating magnetic field on growth rate, cell metabolic activity and biofilm formation by *Staphylococcus aureus* and *Escherichia coli*. J. Magn..

[29] Fijałkowski K., Nawrotek P., Struk M., Kordas M., Rakoczy R. (2015). Effects of rotating magnetic field exposure on the functional parameters of different species of bacteria. Electromagn. Biol. Med..

[30] Fijałkowski K., Drozd R., Żywicka A., Junka A.F., Kordas M., Rakoczy R. (2017). Biochemical and cellular properties of *Gluconacetobacter xylinus* cultures exposed to different modes of rotating magnetic field. Pol. J. Chem. Technol..

[31] Konopacka A., Rakoczy R., Konopacki M. (2018). The effect of rotating magnetic field on bioethanol production by yeast strain modified by ferrimagnetic nanoparticles. J. Magn. Magn. Mater..

[32] Lechowska J., Kordas M., Konopacki M., Fijałkowski K., Drozd R., Rakoczy R. (2019). Hydrodynamic studies in magnetically assisted external-loop airlift reactor. Chem. Eng. J..

[33] Rampersad S.N. (2012). Multiple applications of Alamar Blue as an indicator of metabolic function and cellular health in cell viability bioassays. Sensors.

[34] Drozd R., Rakoczy R., Konopacki M., Frąckowiak A., Fijałkowski K. (2017). Evaluation of usefulness of 2DCorr technique in assessing physicochemical properties of bacterial cellulose. Carbohydr. Polym..

[35] Nelson M.L., O’Connor R.T. (1964). Relation of certain infrared bands to cellulose crystallinity and crystal latticed type. Part I. Spectra of lattice types I, II, III and of amorphous cellulose. J. Appl. Polym. Sci..

[36] Ha S.-J., Kim S.-Y., Seo J.-H., Oh D.-K., Lee J.-K. (2007). Optimization of culture conditions and scale-up to pilot and plant scales for coenzyme Q10 production by *Agrobacterium tumefaciens*. Appl. Microbiol. Biotechnol..

[37] Jung H.-M., Kim S.-Y., Moon H.-J., Oh D.-K., Lee J.-K. (2007). Optimization of culture conditions and scale-up to pilot and plant scales for vancomycin production by *Amycolatopsis orientalis*. Appl. Microbiol. Biotechnol..

[38] Ruka D.R., Simon G.P., Dean K.M. (2012). Altering the growth conditions of *Gluconacetobacter xylinus* to maximize the yield of bacterial cellulose. Carbohydr. Polym..

[39] Wu S.-C., Li M.H. (2015). Production of bacterial cellulose membranes in a modified airlift bioreactor by *Gluconacetobacter xylinus*. J. Biosci. Bioeng..

[40] Reiniati I., Hrymak A.H., Margaritis A. (2017). Kinetics of cell growth and crystalline nanocellulose production by *Komagataeibacter xylinus*. Biochem. Eng. J..

[41] Cheng H.-P., Wang P.-M., Chen J.-W., Wu W.-T. (2002). Cultivation of *Acetobacter xylinum* for bacterial cellulose production in a modified airlift reactor. Appl. Biochem. Biotechnol..

[42] Kongruang S. (2008). Bacterial BC production by *Acetobacter xylinum* strains from agricultural waste products. Appl. Biochem. Biotechnol..

[43] Augimeri R.V., Varley A.J., Strap J.L. (2015). Establishing a role for bacterial cellulose in environmental interactions: Lessons learned from diverse biofilm-producing Proteobacteria. Front. Microbiol..

[44] Park K.J., Jung J.Y., Park Y.H. (2003). Cellulose production by *Gluconacetobacter hansenii* in a medium containing ethanol. Biotechnol. Lett..

[45] Aydin Y.A., Aksoy N.D. (2014). Isolation and characterization of an efficient bacterial cellulose producer strain in agitated culture: *Gluconacetobacter hansenii* P2A. Appl. Microbiol. Biotechnol..

[46] Chawla P., Bajaj I., Shrikant S., Singhal R.S. (2009). Microbial Cellulose: Fermentative Production and Applications. Food Technol. Biotechnol..

[47] Song H.J., Li H., Seo J.H., Kim M.-J., Kim S.-J. (2009). Pilot-scale production of bacterial cellulose by a spherical type bubble column bioreactor using saccharified food wastes. Korean J. Chem. Eng..

[48] Aswini K., Gopal N.O., Uthandi S. (2020). Optimized culture conditions for bacterial cellulose production by *Acetobacter senegalensis* MA1. BMC Biotechnol..

[49] Cheng Z., Yang R., Liu X., Chen H. (2017). Green synthesis of bacterial cellulose via acetic acid pre-hydrolysis liquor of agricultural corn stalk used as carbon source. Bioresour. Technol..

[50] Yanti N.A., Ahmad S.W., Muhiddin N.H. (2018). Evaluation of inoculum size and fermentation period for bacterial cellulose production from sago liquid waste. J. Phys..

[51] Potivara K., Phisalaphong M. (2019). Development and characterization of bacterial cellulose reinforced with natural rubber. Materials.

[52] Treesuppharat W., Rojanapanthu P., Siangsanoh C., Manuspiya H., Ummartyotin S. (2017). Synthesis and characterization of bacterial cellulose and gelatin-based hydrogel composites for drug-delivery systems. Biotechnol. Rep. (Amst.).

[53] Ul Islam M., Khan T., Kon Park J.K. (2012). Water holding and release properties of bacterial cellulose obtained by in situ and ex situ modification. Carbohydr. Polym..

[54] Tsouko E., Kourmentza C., Ladakis D., Kopsahelis N., Mandala I., Papanikolaou S., Paloukis F., Alves V., Koutinas A. (2015). Bacterial cellulose production from industrial waste and by-product streams. Int. J. Mol. Sci..

[55] Ciecholewska-Juśko D., Broda M., Żywicka A., Styburski D., Sobolewski P., Gorący K., Migdał P., Junka A., Fijałkowski K. (2021). Potato juice, a starch industry waste, as a cost-effective medium for the biosynthesis of bacterial cellulose. Int. J. Mol. Sci..

[56] Tokoh C., Takabe K., Fujita M., Saiki H. (1998). Cellulose synthesized by *Acetobacter xylinum* in the presence of acetyl glucomannan. Cellulose.

[57] Kaminski K., Jarosz M., Grudzien J., Pawlik J., Zastawnik F., Pandyra P., Kołodziejczyk M.A. (2020). Hydrogel bacterial cellulose: A path to improved materials for new eco-friendly textiles. Cellulose.

[58] Stanisławska A., Staroszczyk H., Szkodo M. (2020). The effect of dehydration/rehydration of bacterial nanocellulose on its tensile strength and physicochemical properties. Carbohydr. Polym..

[59] Chen S.Q., Lopez-Sanchez P., Wang D., Mikkelsen D., Gidley M.J. (2018). Mechanical properties of bacterial cellulose synthesised by diverse strains of the genus *Komagataeibacter*. Food Hydrocoll..

[60] McKenna B.A., Mikkelsen D., Wehr J.B., Gidley M.J., Menzies N.W. (2009). Mechanical and structural properties of native and alkali-treated bacterial cellulose produced by *Gluconacetobacter xylinus* strain ATCC 53524. Cellulose.

[61] Cielecka I., Ryngajłło M., Bielecki S. (2020). BNC Biosynthesis with increased productivity in a newly designed surface air-flow bioreactor. Appl. Sci..

[62] Krystynowicz A., Czaja W., Wiktorowska-Jezierska A., Gonçalves-Miśkiewicz M., Turkiewicz M., Bielecki S. (2002). Factors affecting the yield and properties of bacterial cellulose. J. Ind. Microbiol. Biotechnol..

[63] Kruer-Zerhusen N., Cantero-Tubilla B., Wilson D.B. (2018). Characterization of cellulose crystallinity after enzymatic treatment using Fourier transform infrared spectroscopy (FTIR). Cellulose.

[64] Czaja W., Romanovicz D., Brown R. (2004). Structural investigations of microbial cellulose produced in stationary and agitated culture. Cellulose.

[65] Manoukian O.S., Sardashti N., Stedman T., Gailiunas K., Ojha A., Penalosa A., Mancuso C., Hobert M., Kumbar S.G. (2019). Biomaterials for Tissue Engineering and Regenerative Medicine. Encyclopedia of Biomedical Engineering.

[66] Lin W.C., Lien C.C., Yeh H.J., Yu C.M., Hsu S.H. (2013). Bacterial cellulose and bacterial cellulose–chitosan membranes for wound dressing applications. Carbohydr. Polym..

[67] Zhijiang C., Guang Y. (2011). Bacterial cellulose/collagen composite: Characterization and first evaluation of cytocompatibility. J. Appl. Polym. Sci..

[68] Sulaeva I., Henniges U., Rosenau T., Potthast A. (2015). Bacterial cellulose as a material for wound treatment: Properties and modifications. A review. Biotechnol. Adv..

