# Supplementary material for: Preparation of Komagataeibacter xylinus Inoculum for Bacterial Cellulose Biosynthesis Using Magnetically Assisted External-Loop Airlift Bioreactor
Source: Polymers (Basel). 2021 Nov 15;13(22):3950. doi: 10.3390/polym13223950 (PMC8623894; doi:10.3390/polym13223950)
Supplement: Supplementary file 1 [file polymers-13-03950-s001.zip › polymers-1441476-supplementary.pdf]

Supplementary Material

Preparation of *Komagataeibacter xylinus* inoculum for bacterial cellulose biosynthesis using magnetically assisted external-loop airlift bioreactor

Anna Żywicka[1,], Daria Ciecholewska-Juśko[1], Radosław Drozd[1], Rafał Rakoczy[2], Maciej Konopacki[2], Marian Kordas[2], Adam Junka[3], Paweł Migdał[4], Karol Fijałkowski[1]

[1]Department of Microbiology and Biotechnology, Faculty of Biotechnology and Animal Husbandry, West Pomeranian University of Technology in Szczecin, Piastów Ave. 45, 70-311 Szczecin, Poland. anna.zywicka@zut.edu.pl; daria.ciecholewska@zut.edu.pl; radoslaw.drozd@zut.edu.pl; karol.fijalkowski@zut.edu.pl;

[2]Department of Chemical and Process Engineering, Faculty of Chemical Technology and Engineering, West Pomeranian University of Technology in Szczecin, Piastów Ave. 42, 71-065 Szczecin, Poland. rafal.rakoczy@zut.edu.pl; maciej.konopacki@zut.edu.pl; marian.kordas@zut.edu.pl

[3]Department of Pharmaceutical Microbiology and Parasitology, Faculty of Pharmacy, Medical University of Wroclaw, Borowska 211a, 50-534 Wrocław, Poland. adam.junka@umed.wroc.pl

[4] Department of Environment, Hygiene and Animal Welfare, Faculty of Biology and Animal Science, Wroclaw University of Environmental and Life Sciences, Chełmońskiego 38C, 51-630 Wrocław, Poland. pawel.migdal@upwr.edu.pl

* Correspondence: AZ: anna.zywicka@zut.edu.pl; +48 91-449-6709; KF: karol.fijalkowski@zut.edu.pl; + 48 91-449-6714.

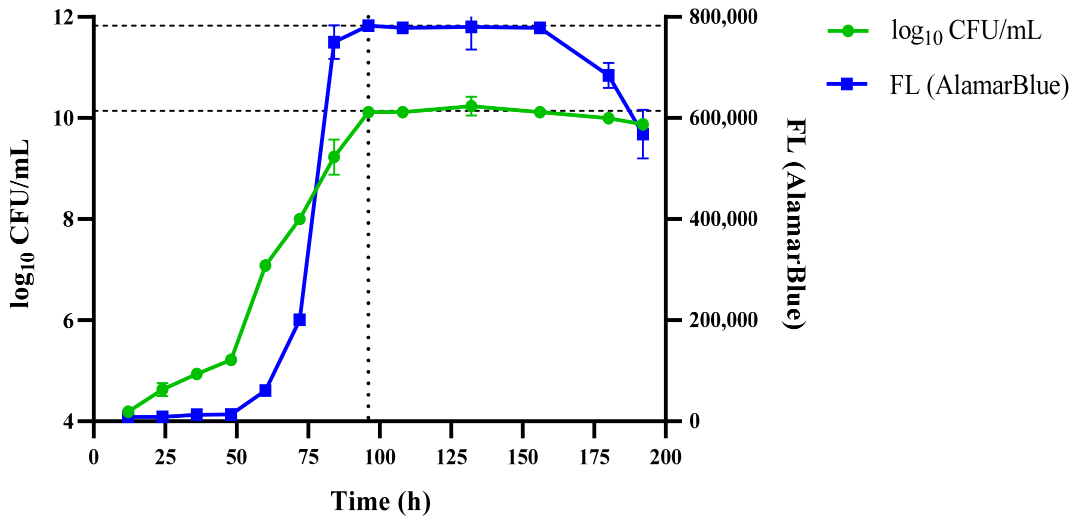

Figure S1. The number of living cells and metabolic activity of *K. xylinus* based on fluorescence of AlamarBlue reagent. Data are presented as a mean ± standard error of the mean (SEM).

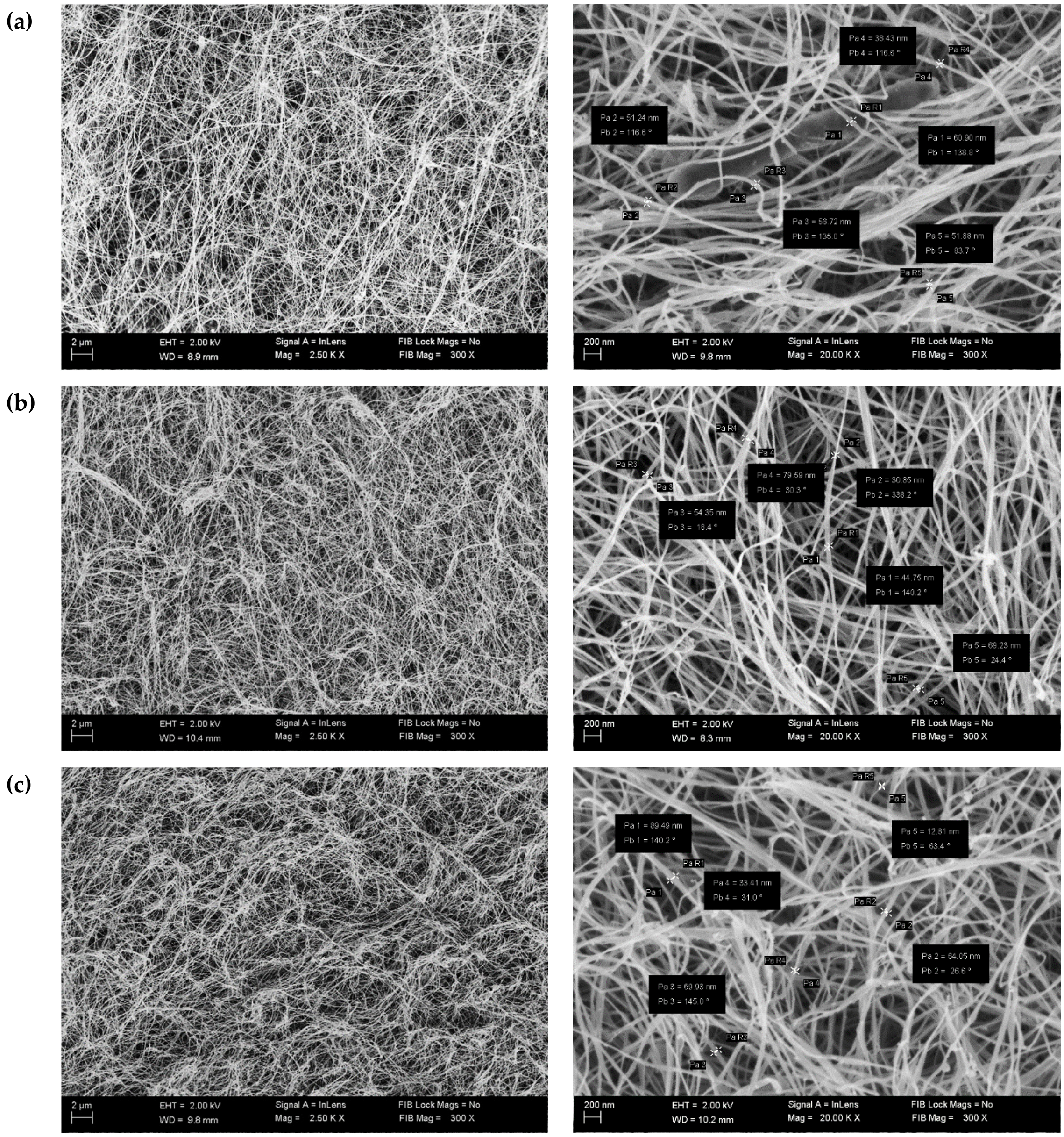

Figure S2. Nanostructure of BC obtained using inoculum produced in RMF-assisted EL-ALB after **(a)** I, **(b)** II, and **(c)** III cycle of fermentation.

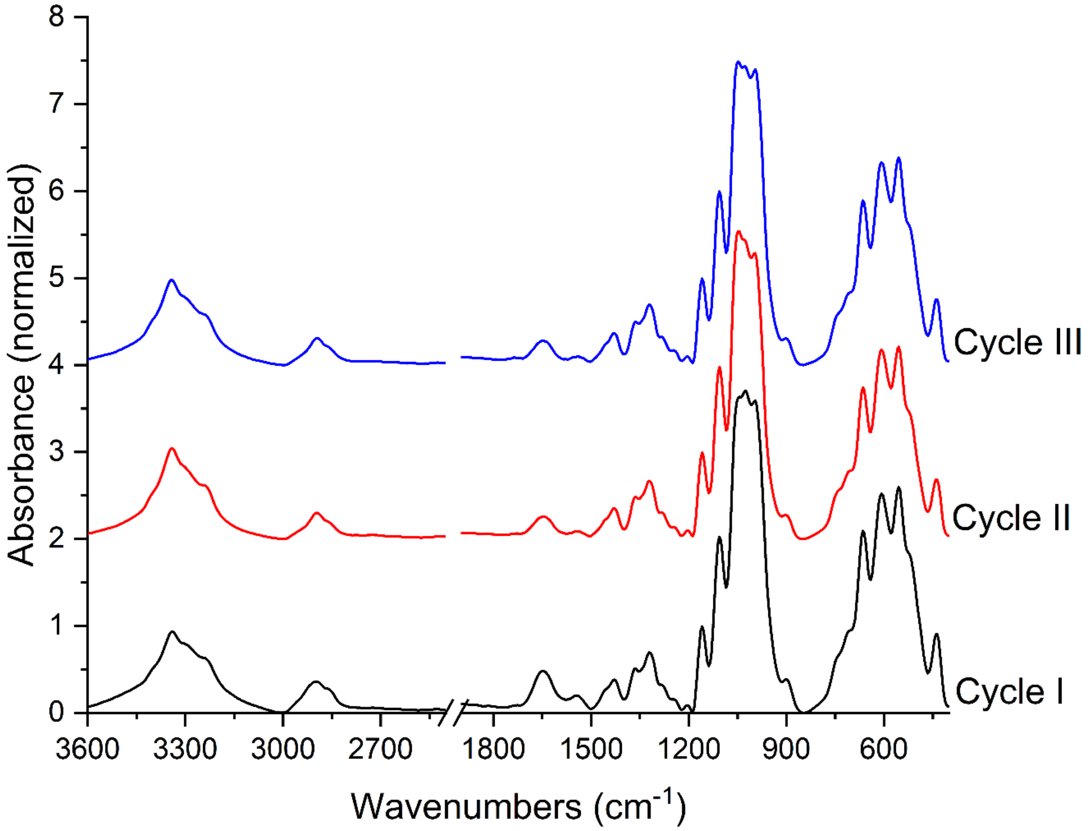

Figure S3. ATR-FTIR spectra of BC obtained using inoculum produced in RMF-assisted EL-ALB after I, II, and III cycle of fermentation. The spectra were processed by baseline correction, and normalization by area of band at 1160 cm^{-1}. The obtained spectra present typical band pattern for ATR-FTIR spectra of BC.
